# qPCR‐based quantification reveals high plant host‐specificity of endophytic colonization levels in leaves

**DOI:** 10.1002/ajb2.16448

**Published:** 2024-12-16

**Authors:** Caio César Pires de Paula, Jiří Bárta, Jakub Borovec, Jan Frouz, Pavel Rychtecký, Dagmara Sirová

**Affiliations:** ^1^ Biology Centre CAS Institute of Hydrobiology Na Sádkách 702/7 České Budějovice 37005 Czech Republic; ^2^ University of South Bohemia Faculty of Science, Branišovská 1645/31a České Budějovice 37005 Czech Republic; ^3^ Institute for Environmental Studies, Faculty of Science Charles University Benátská 2, CZ‐ 12800 Prague Czech Republic

**Keywords:** cell counts, ecological succession, foliar endophyte, fungi‐bacteria ratios, qPCR, soil chronosequence

## Abstract

**Premise:**

Despite the high functional importance of endophytes, we still have limited understanding of the biotic and abiotic factors that influence colonization of plant hosts along major ecological gradients and lack quantitative estimates of their colonization extent. In this study, we hypothesized that the developmental stage of the ecosystem will affect the levels of bacterial and fungal endophytic assemblages in the foliar endosphere.

**Methods:**

We quantified levels of bacterial and fungal endophytes in leaves of four plant hosts at four stages of vegetation succession using an optimized qPCR protocol with bacteria‐specific 16S and fungi‐targeting primers.

**Results:**

(1) The ecosystem developmental stage did not have a significant effect on the colonization levels of bacterial or fungal endophytes. (2) Colonization levels by bacterial and fungal endophytes were governed by different mechanisms. (3) Endophytic colonization levels and their relationship to foliar tissue stoichiometry were highly host specific.

**Conclusions:**

Quantifying colonization levels is important in the study of endophytic ecology, and the fast, relatively low‐cost qPCR‐based method can supply useful ecological information, which can significantly enhance the interpretation potential of descriptive data generated, for example, by next‐generation sequencing.

## INTRODUCTION

Endophytes inhabit the leaves of species in all currently described plant phyla (Harrison and Griffin, 2020). They represent species‐rich and phylogenetically diverse assemblages of mainly bacteria and fungi worldwide, across every terrestrial biome (Apigo and Oono, 2018, 2022). Endophytic community composition and function have become important areas of research within the field of plant–microbe interactions, with a rapidly increasing number of published studies (for review, see Harrison and Griffin, 2020). While we are starting to have a good idea about endophytic diversity and general assemblage structure, we lack quantitative information on the assemblages within the plants. The level of endophytic colonization can be an important metric in studies of plant–microbe interactions—serving as a proxy for the assemblage‐level growth, turnover, and biomass of endophytes, as well as nutrient availability or the availability of space for colonization within the endosphere. Some of the present questions include the following: Are the leaves of different plant species colonized to a different extent by endophytic microbes? How does the abundance of endophytes change over the seasons? Does ecosystem spatial heterogeneity affect endophytic levels? Do bacteria and fungi have similar overall patterns of colonization, or are they affected by different factors? Even though answers to these questions would help advance research of their ecology and interactions, quantitative data are only rarely obtained in studies involving endophytic microbes.

One of the reasons for this lack of quantitative data may be the difficulty in quantifying bulk endophytes in plant tissues. Methods such as microscopy, counts of colony‐forming units, or the analysis of organism‐specific biomolecules are laborious and notoriously unreliable, often requiring large sample sizes and frequently lacking coverage of major prokaryotic and fungal lineages (Olsson et al., 2003).

Real‐time quantitative PCR (qPCR) using 16S or 18S and ITS rDNA primers is a well‐established method for the detection and quantification of bacteria and fungi, respectively, in ecological studies. The qPCR method combines end‐point detection PCR with fluorescence detection to track the accumulation of amplicons during each amplification cycle (Smith and Osborn, 2009). By detecting amplicons during the early exponential phase of the PCR, in proportion to the starting template concentration, it is possible to quantify gene copy numbers in a sample (Smith and Osborn, 2009); however, qPCR of microbes in plant tissue samples remains problematic. Several studies have used qPCR to quantify bacterial/archaeal endophytic colonization (e.g., Müller et al., 2015), but they did not address the key issue of contamination by host organellar (particularly chloroplast) DNA, which can, in our experience, result in an overestimation of up to several orders of magnitude caused by the high affinity of the universal bacterial 16S rDNA primer pairs for nontarget DNA. In fungi, on the other hand, the most widely used rDNA targets for designing qPCR assays are present in multiple copies (in the tens to hundreds) in the genome, with high intraspecific variation frequent in many of the now fully sequenced fungi (Lofgren et al., 2018), thus limiting the usability of these primers for reliable quantification (Black et al., 2013).

Here, we attempted to overcome the pitfalls associated with qPCR quantification of the total levels of bacteria and fungi in the foliar endosphere by using approaches with greater target specificity and therefore greater utility in field ecological studies. The bacterial qPCR protocol used here is based on primer set 335F/769R with high reported specificity for bacterial DNA (Dorn‐In et al., 2015) and good performance in next‐generation 16S rDNA amplicon sequencing of plant tissue samples (Nakano, 2018). The qPCR protocol for the quantification of fungi is based on a primer set targeting the fungi‐specific β‐actin gene (Carbone and Kohn, 1999; Ettenauer et al., 2014). Fungi appear to have a tendency toward a single actin gene copy per haploid genome, and the majority of vegetative fungal biomass in nature is presently assumed to be in a haploid state (e.g., Nieuwenhuis and James, 2016; Tyrrell et al., 2020). Therefore, the β‐actin gene copy number determined by qPCR should represent the number of fungal nuclei in a given plant tissue sample. While the number of nuclei is not directly translatable to biomass of fungi, which often exist in a dikaryotic state (hyphal cells contain two compatible nuclei that do not undergo nuclear fusion) or do not consist of individual cells but rather form hyphae as continuous compartments with numerous migrating nuclei (Gladfelter and Berman, 2009), each nucleus does require a specific minimal volume of cytoplasm (Anderson et al., 2013). Therefore, a positive relationship should exist between the number of nuclei and fungal biomass in a specific sample (Cantwell and Nurse, 2019). The use of β‐actin as a qPCR target thus should enable relatively precise quantification of fungal endophytic colonization extent for cross‐sample comparisons.

After experimentally verifying this assumed relationship by comparing microscopy‐based fungal nuclei counts with numbers generated by qPCR, we assessed the variation in the colonization extent of foliar endophytic bacteria and fungi in four phylogenetically distant plant species growing along a vegetation successional gradient at three times during the growing season. Our study area was located within the unreclaimed areas of large colliery spoil heaps formed by open‐cast coal mining in the northwestern part of the Czech Republic. The experimental locations are covered by vegetation in four successional stages in a continuous soil chronosequence, which allowed us to study the effect of ecosystem heterogeneity, plant host identity, and sampling time on the endophytic levels at sites having similar parent material, climate, and regional species pools (Frouz et al., 2008; Mudrák et al., 2016). The specific phases of ecosystem development have been well documented at our long‐term study sites, revealing strong and predictable shifts in plant community composition, soil microbial community composition (Frouz and Nováková, 2005), overall ecosystem biodiversity, and ecosystem processes such as primary productivity, biomass accumulation, or soil nutrient cycling and decomposition (Harantová et al., 2017).

We know from the literature that many plant host traits important for competitive success at an early stage of succession usually differ significantly from those that prevail at later stages (e.g., Lambers et al., 2008). Those related to leaves include area‐based nutrient content (Bazzaz, 1979; Lambers et al., 2008). Although published information on the effect of leaf traits on endophytes is scarce, the effect is assumed to be significant (Tellez et al., 2022). Because leaf nutrient content has a significant effect on the structuring of bacterial endophytic assemblages (Borruso et al., 2018), we chose leaf stoichiometry as a proxy for characterizing the differences between the experimental host plant species growing at different stages of ecosystem development and hypothesized that ecosystem age will play a significant role in shaping colonization levels of foliar endophytes.

## MATERIALS AND METHODS

### Verification of 16S primer specificity for bacterial rDNA

Bacterial 16S rDNA counts were determined in test samples using two different primer pairs for comparison: (1) the widely used set of primers targeting bacteria, recommended by the Earth Microbiome Project (https://earthmicrobiome.org/protocols-and-standards/16s/; e.g., Walters et al., 2016; 515F: 5′‐GTGYCAGCMGCCGCGGTAA‐3′/806R: 5′‐GGACTCANVGGGTWTCTAAT‐3′), which was suspected to co‐amplify plant organellar DNA and (2) primer pair 335F/769R (5′‐CADACTCCTACGGGAGGC‐3′/5′‐ATCCTGTTTGMTMCCCVRRC‐3′) with reported high specificity for bacterial DNA only (Dorn‐In et al., 2015). To test the performance of the two primer pairs in samples with varying DNA concentrations and different ratios of bacterial to organellar DNA, we used the ZymoBIOMICS Microbial Community DNA Standard II (Zymo Research, Irvine, CA, USA; consisting of genomic DNA from 8 defined bacterial and 2 defined fungal strains) and genomic DNA from an axenic culture of *Chlorella vulgaris* (UTEX 2714; Culture Collection of Algae, University of Texas, Austin, TX, USA) to prepare nine samples as follows: two mock community and two algal genomic DNA (gDNA samples with two different DNA concentrations (0.1 and 0.01 ng DNA µL^–1^) for each sample type, and five mixed samples with different proportions of mock community to algal gDNA (100:1 [1.01 ng DNA µL^–1^], 10:1 [1.10 ng DNA µL^–1^], 1:1 [2.0 ng DNA µL^–1^], 1:10 [1.10 ng DNA µL^–1^], 1:100 [1.01 ng DNA µL^–1^]). 16S rDNA gene copies were quantified in triplicate samples using qPCR (LightCycler 96 system) with 10 μL FastStart Essential DNA Green Master 2× concentrated (Roche Molecular Biochemicals, Mannheim, Germany) and 1.0 μM of each primer. Cycling conditions were specific for each primer set: 335F/769R (10 min at 95°C; 40 cycles: 10 s at 95°C, 15 s at 60°C, 30 s at 72°C; melting phase: 10 s at 95°C, 60 s at 60°C, 1 s at 95°C, 30 s at 37°C) and 515F/806R (95°C for 10 min; 40 cycles of 95°C for 15 s, 60°C for 30 s, 72°C for 30 s, melting stage of 95°C for 10 s, 60°C for 60 s, 95°C for 1 s; 37°C for 30 s). The cycle quantification (Cq) values were converted to the number of target gene copies according to the standard curves generated from cloned targets (10‐fold serial dilutions from 10^–1^ to 10^–4^). The results were expressed as the number of gene copies per nanogram of total extracted DNA.

### Comparison of the performance of fungal qPCR primers and microscopic fungal cell counts

We used a standardized mock community with varying proportions of different organisms to compare the performance of three fungal gene targets commonly used in PCR (ITS, 18S, and β‐actin) with direct microscopic enumeration of fungal cells (nuclei). We selected three yeast species reported previously to be present in the plant endosphere, growing axenically in haploid form (*Pichia kudriavzevii* strain CCM8271, *Cyberlindnera jadinii* strain CCM8188, and *Meyerozyma quilliermondii* strain CCM8321 from the Czech Collection of Microorganisms, CCM), a bacterium (*Escherichia coli* strain CCM3954), and a single‐celled green alga (*Monoraphidium contortum* from the algal collection of the Institute of Hydrobiology, Biology Centre CAS). The yeast strains were cultivated on glucose‐peptone‐yeast extract agar (GPYA: glucose 40.0 g L^–1^, peptone 5.0 g L^–1^, yeast extract 5.0 g L^–1^, pH 5.6). *E. coli* was grown in Nutrient Broth (Merck) and *M. contortum* on WC agar (Guillard and Lorenzen, 1972). All strains were incubated at 25°C for 3 days. The *P. kudriavzevii* culture formed short pseudohyphae; the remaining two yeast strains grew as single cells. Before DNA extraction, the cultures were diluted to a standard concentration of 1 × 10^7^ cells mL^–1^. Six mock communities were created by combining the cells of the above five strains in different proportions (Table 1). Triplicate formaldehyde‐fixed subsamples were used to count yeast cells using an epifluorescence microscope (BX53; Olympus, Tokyo, Japan) after DAPI (4′,6‐diamidino‐2‐phenylindole) staining of cell nuclei, according to standard procedures (Šimek and Sirová, 2019). The remaining subsample was used for DNA extraction: a total of 0.5 mL per dilution was centrifuged (10,000 rpm; 10 min) to pellet the cells. The Quick‐DNA Fecal/Soil Microbe Miniprep Kit (Zymo Research) was used to extract DNA from the pellets according to the manufacturer's protocols. Qubit 3.0 fluorometer (Life Technologies, Carlsbad, CA, USA) and NanoDrop 2000 Spectrophotometer (Thermo Fisher Scientific, Waltham, MA, USA) were used to verify the DNA quantity and quality, respectively. Gene copy numbers were estimated using the LightCycler 96 system (Roche Molecular Biochemicals, Mannheim, Germany) equipped with the LightCycler DNA Master SYBR Green I (Roche). We used primers ITS1/ITS2 (5′‐TCCGTAGGTGAACCTGCGG‐3′/5′‐TGCTGCGTTCTTCATCGATGC‐3′), nu_SSu_1196/nu_SSu_0817 (5′‐TCTGGACCTGGTGAGTTTCC‐3′‐TTAGCATGGAATAATRRAATAGGA), and ACT‐512F/ACT‐783R (5′‐ATGTGCAAGGCCGGTTTCGC‐3′/5′‐TACGAGTCCTTCTGGCCCAT‐3′) with specific cycling conditions for each gene target (ITS: 10 min at 95°C; 40 cycles: 30 s at 95°C, 30 s at 58°C, 45 s at 72°C; melting phase: 10 s at 95°C, 60 s at 58°C, 10 s at 95°C; 30 s at 37°C; 18S: 10 min at 95°C; 40 cycles: 30 s at 95°C, 30 s at 56°C, 45 s at 72°C; melting phase: 10 s at 95°C, 60 s at 56°C, 10 s at 95°C; 30 s at 37°C; β‐actin: 10 min at 95°C; 40 cycles: 30 s at 95°C, 30 s at 50°C, 45 s at 72°C; melting phase: 10 s at 95°C, 60 s at 50°C, 10 s at 95°C; 30 s at 37°C). The Cq values were converted to copy numbers of the gene target per nanogram of DNA, using standard curves generated from cloned targets (10‐fold serial dilutions from 10^–1^ to 10^–4^) as a reference, and the values were converted to number of nuclei per milliliter according to the initial volume of sample used for DNA extraction, for direct comparison with cell counts. The specificity of the amplification products was confirmed by melting‐curve analysis and gel electrophoresis (Appendix S1). Because β‐actin is present as a single copy in the haploid fungal genome, we expected to see, contrary to the multicopy ITS and 18S targets, a tight match for each sample between gene copy numbers and the direct counts of yeast nuclei.

**Table 1:** Composition of standardized mock communities used for the comparison of qPCR‐based quantification of fungal gene copy numbers with direct microscopy‐based cell counts.

Mock community	*Pichia kudriavzevii*, *Cyberlindnera jadinii*, and *Meyerozyma quilliermondii* (1:1:1)	*Escherichia coli*, and *Monoraphidium contortum* (1:1)
MC1	100%	0%
MC2	80%	20%
MC3	60%	40%
MC4	40%	60%
MC5	20%	80%
MC6	0%	100%

### Quantification of endophytic colonization in leaf samples from the experimental sites

#### Study site

The study area (50°14′21″ N, 12°40′45″ E) included four unreclaimed post‐mining sites formed by alkaline tertiary clay, which was deposited during lignite mining, near Sokolov, Czech Republic, 500–600 m a.s.l., with an average annual precipitation of 650 mm and a mean annual temperature of 6.8°C. Because of the relatively high amount of fossil organic matter and mild climate (Urbanová et al., 2011), the heaps represent an opportunity to follow primary succession processes in a non‐extreme environment.

The sites are overgrown by vegetation in various stages of spontaneous succession. We selected four plant species with distinct phylogeny and ecophysiology for sampling leaf endophytes: a perennial grass [*Calamagrostis epigejos* (L.) Roth, Poaceae], a deciduous tree/shrub (*Salix caprea* L., Salicaceae), a coniferous tree (*Picea abies* L., Pinaceae), and a perennial herb (*Tussilago farfara* L., Asteraceae). All four species are present at all four sites (10, 20, 30, and 54 years after clay deposition, designated here as I, II, III, and IV, respectively) where the vegetation changes from herb‐dominated at the youngest location through shrub‐dominated to forest‐dominated at the oldest site (Frouz et al., 2008). *Calamagrostis epigejos* dominated at the two youngest sites, interspersed with *S. caprea* and *P. abies* individuals, which here reached only shrub height. The 30‐year‐old site harbored a transitional forest with grown *S. caprea* trees as dominants and a sparse population of the other three species. The oldest site contained a developed forest in which the dominant trees were *Betula pendula* and *Populus tremula*, while *C. epigejos* dominated the understory. *Salix caprea* and *P. abies* remained sparse.

#### Plant material

In the first week of May 2017 (spring, start of growing season), six individuals, growing at least 15 m apart, from each species and each location were selected for sampling and marked. Two to six individual leaves without visible damage or disease, depending on the plant, were collected for *S. caprea*, *T. farfara*, and *C. epigejos*. The needles from four branches from the growth of 2016 were collected for *P. abies*. For the two tree species, leaves with petioles were collected from different sides of the tree at the same height from the ground. To ensure that the true endophytic community was analyzed, we processed the leaves in the field using the protocol of Fischer et al. (1993) with the following modifications: The leaves were thoroughly washed in tap water and surface‐sterilized in 75% v/v ethanol for 1 min, 0.5 M sodium hypochlorite for 3 min, and 75% v/v ethanol for 0.5 min, with a final wash in Milli‐Q Water (MilliporeSigma water system, Merck KGaA, Darmstadt, Germany) and placed immediately into liquid nitrogen. The efficacy of surface sterilization was tested periodically by stamping a sterilized leaf onto an agar plate and determined to be negligible. The viability of endophytes after surface sterilization was confirmed by plating sterilized leaf homogenates and isolating endophytic strains (data not shown). After transport to the laboratory, pooled leaf samples from a single individual were finely ground in liquid nitrogen using a sterile mortar and pestle and kept at –80°C until further analysis (tissue chemistry and DNA extraction). The same individuals were sampled again the first week of August (summer, peak of growing season) and the second week of October 2017 (autumn, beginning of senescence).

#### DNA extraction and qPCR

Approximately 100 mg of homogenized, ground leaf sample was used for DNA extraction using the DNeasy Plant Mini Kit (Qiagen GmbH, Hilden, Germany) according to the manufacturer's instructions. The quantity and quality of DNA extracts were determined using the Qubit 3.0 fluorometer and NanoDrop 2000 Spectrophotometer, respectively. Bacterial and fungal endophytic colonization in field‐collected leaf samples was quantified using qPCR and the LightCycler 96 system (Roche, Basel, Switzerland). Bacterial counts were estimated using the bacteria‐specific 16S rDNA primer 335F/769R (Dorn‐In et al., 2015), and the β‐actin target gene (primer pair ACT‐512F/ACT‐783R) was used to quantify endophytic fungi (Ettenauer et al., 2014). The qPCR protocol used for each target gene is described in detail above. Four standard curves per target region (16S and β‐actin genes) were obtained using 10‐fold serial dilutions (10^–1^ to 10^–4^) of plasmids generated from cloned targets. Results were normalized to represent the average copy number of targets per ng of DNA, due to large variation in the amount of extracted DNA within the data set (Appendix S2).

### Leaf chemistry analyses

Because C, N, and P are among the quantitatively most important essential elements and in general expected to limit both plant and microbial growth (Keiblinger et al., 2010), we analyzed C, N, and P content in each bulked leaf sample as a proxy for the leaf environment colonized by foliar endophytes. Total C and total N contents in homogenized and freeze‐dried leaf tissue were analyzed using the CHNS analyzer Vario MICRO Cube (Elementar, Langenselbold, Hesse, Germany). Total P content was measured using the triple quadrapole ICP‐QQQ tandem mass spectrometer (Agilent Technologies, Santa Clara, California, USA) after wet digestion with HClO_4_ (at 170°C for 2 h). Results were expressed in milligrams per milligram of dry leaves.

### Statistical analyses

Statistical analyses were carried out using R (version 4.0.2; R Core Team, 2020) and Past 4.14 (Hammer et al., 2001). Values were log‐transformed (base 10) to account for data variability and allow comparison among different samples. The results were analyzed using basic descriptive statistics (Shapiro–Wilks), coefficient of variation (CV), or Student's *t*‐test with a 5% probability threshold in Past software. Linear regression was used to evaluate the accuracy of the primers tested in relation to the standard communities and the ratio of bacterial and fungal quantity, and plots were generated using the R package ggplot2 (v3.5.1, Wickham, 2016). Permutational multivariate analysis of variance (PERMANOVA, number of permutations = 999) was used to test the effect of the sampling groups (plant species; ecosystem development stage; season) using the function adonis in the R package vegan (v2.5.6; Oksanen et al., 2014). Pearson's correlation analysis was used to verify the relationship between the bacterial and fungal quantity and stoichiometry data of plant tissue.

## RESULTS

### Performance of 16S rDNA primer pairs 515F/806R and 335F/769R in quantifying bacteria in the presence of plant organellar DNA

**Figure 1:**
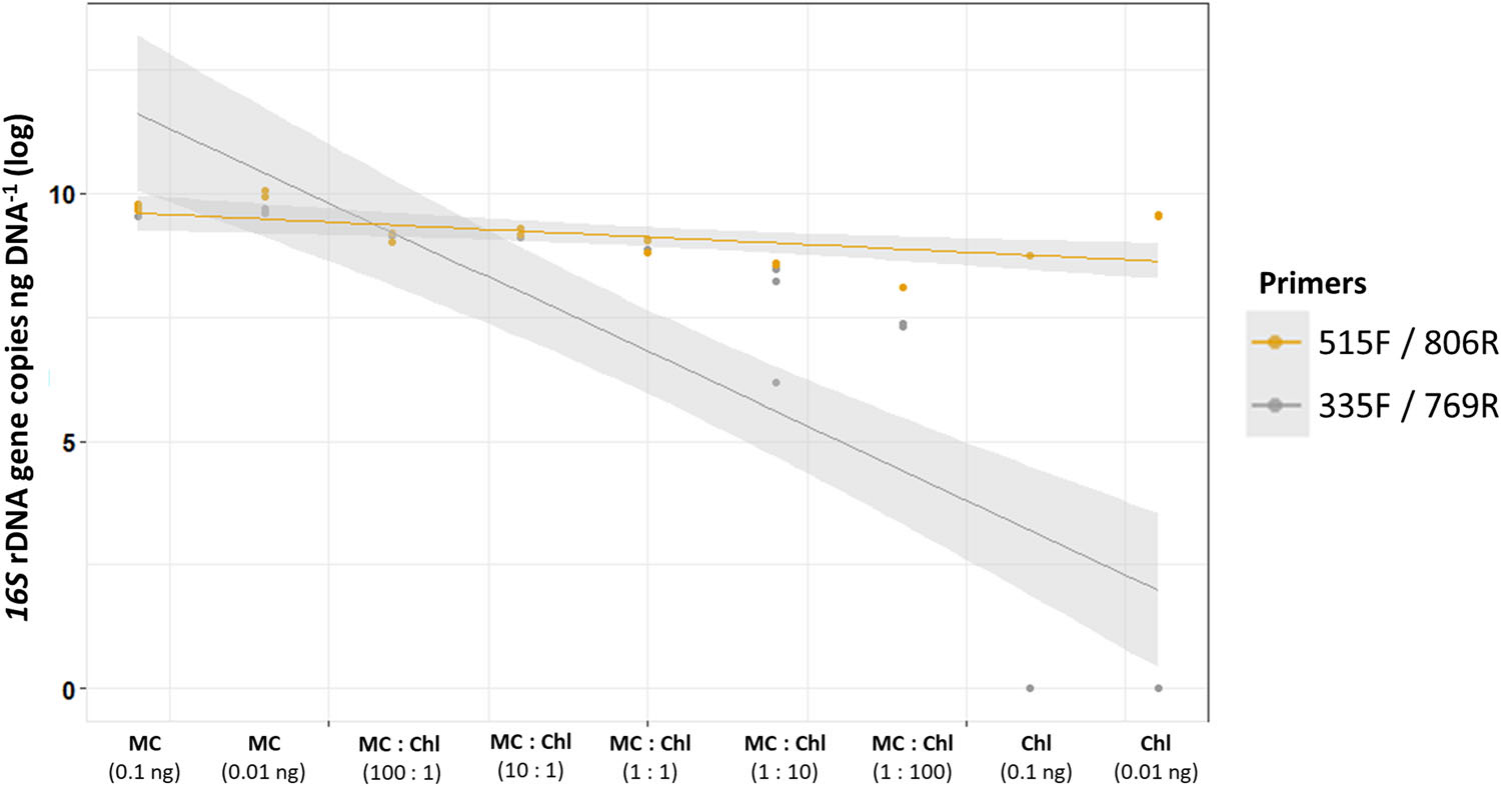
Bacterial quantification (no. 16S rDNA copies ng DNA^–1^) using qPCR with primer set 515 F/806 R (yellow) or 335 F/769 R (gray). Nine sample compositions in two dilutions each were tested: mock community sample of both bacterial and fungal genomic DNA (MC; 0.1 and 0.01 ng DNA µL^–1^), *Chlorella vulgaris* genomic DNA from an axenic culture (Chl; 0.1 and 0.01 ng DNA µL^–1^), and mixed samples with 100:1, 10:1, 1:1, 1:10, or 1:100 of the mock community and algal genomic DNA (MC:Chl). Values were log‐transformed (base 10), shaded area represents the 95.0% confidence interval.

We compared primer pair performance in the presence of plant organellar DNA, as described in detail above, in nine sample types with different concentrations of defined bacterial mock community DNA and algal genomic DNA. Results were log‐transformed (base 10) so that the differences were visible (Figure 1). In general, the widely used bacterial primer set 515F/806R yielded an overestimation of the bacterial counts of up to six times, compared to the values obtained using primer pair 335F/769R (Appendix S3). Because samples containing only algal DNA were amplified by the 515F/806R primer pair, but not by 335F/769R, we attribute this overestimation to co‐amplification of nontarget algal chloroplast and mitochondrial DNA, and, in samples where the algal DNA was absent, to co‐amplification of nontarget fungal mitochondrial DNA. The bacteria‐specific primer pair 335F/769R also offered the advantage of overall lower data variability (CV = 3.96 ± 3.56%) and, hence, increased quantification precision, compared to primer set 515F/806R (CV = 28.37 ± 30.41%).

### Performance of fungi‐specific β‐actin primer in quantifying fungi using qPCR, verified by microscopy

**Figure 2:**
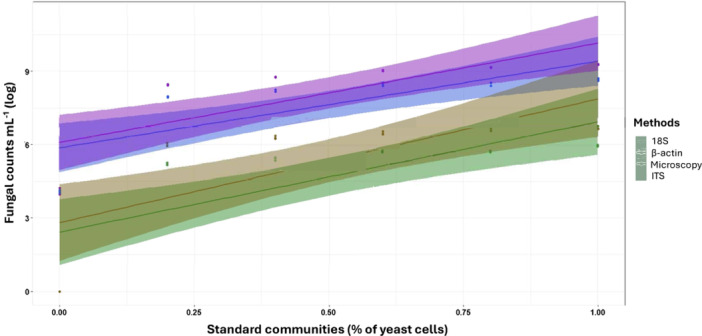
Accuracy of quantifying individual cells in a standardized mock community consisting of three yeast strains, a bacterium, and a green alga, using direct counts with fluorescence‐microscopy and qPCR with three target fungal genes (18S, ITS, and β‐actin). Samples of mock communities with different proportions of the five organisms were quantified using both approaches. Microscopy counts (brown line) were based on DAPI staining of nuclei. Primers for qPCR quantification nu_SSu_1196/nu_SSu_0817 for 18S (blue line), ITS1/ITS2 for ITS (pink line), and ACT‐512F/ACT‐783R for β‐actin (green line). Values were log‐transformed (base 10), and the shaded area represents the 95.0% confidence interval.

Copy number results for all three primer sets (specific for ITS, 18S, or β‐actin) showed collinearity with the proportion of yeast cells in each standard community and with the cell counts (Figure 2; Appendix S4). Although the same trend was observed for all amplified fragments, only the β‐actin gene copy numbers were not statistically different from the yeast nuclei numbers estimated by microscopy (6.0 × 10^2^ to 9.8 × 10^5^ cells mL^–1^, *P* = 0.63). In contrast, the 18S and ITS gene copy numbers showed overestimation errors of several orders of magnitude and large variability. The worst result was observed for ITS (9.2 × 10^3^ to 1.9 × 10^9^ cells mL^–1^, *P* = 0.0005) with qPCR quantification values up to 425.9 times larger than the cell counts. The 18S gene copy numbers (1.3 × 10^4^ to 3.9 × 10^8^ cells mL^–1^, *P* = 0.002) showed overestimation errors of 64.7 to 124.6 times, compared to the cell counts. The β‐actin primer set performed well, as can be seen in Figure 2, where the qPCR linear regression model falls fully within the 95% confidence interval of yeast cell counts estimated.

### Levels of endophytes in leaves from experimental sites differing in ecosystem development stage

#### Bacterial endophytes

**Figure 3:**
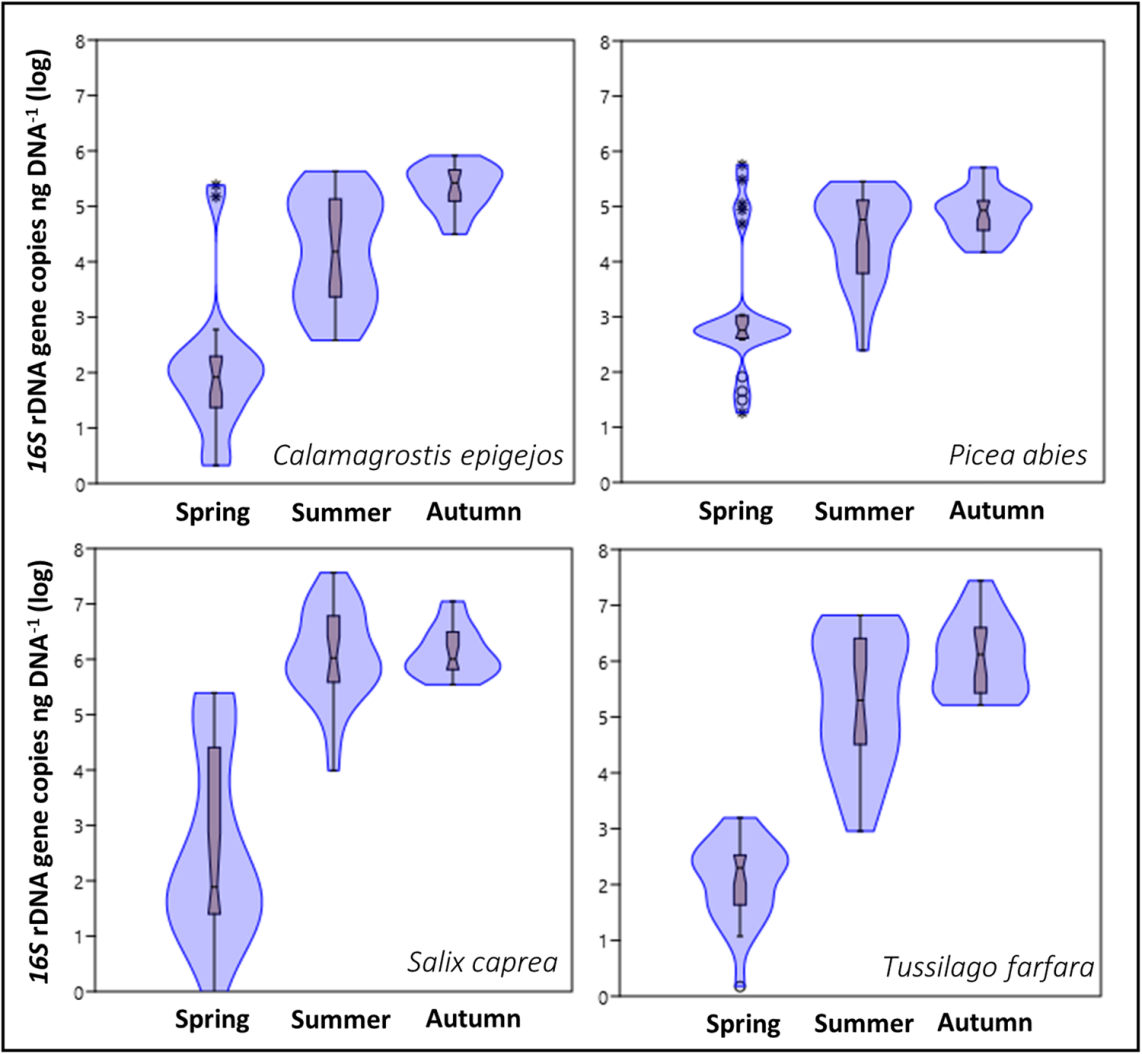
Violin plots showing the level of bacterial endophytes (as 16S rDNA gene copies ng DNA^–1^) in leaves from four plant host species (*Calamagrostis epigejos*, *Picea abies*, *Salix caprea*, and *Tussilago farfara*) from the experimental locations along a chronosequence gradient, Sokolov, Czech Republic, at three sampling times in the growing season (spring, summer, and autumn). Values were log‐transformed (base 10); box plots display the median (central constriction), interquartile range (upper and lower box borders); lower/upper adjacent values (black lines), and outliers (open circles and stars).

In general, bacterial 16S rDNA copy numbers in the leaf endosphere ranged between 0.86 and 35.6 × 10^6^ copies per ng DNA, with high variability among individuals of the same species at a given location and time (Appendix S2). While the location or successional stage had no significant effect (*F*_3,272_ = 0.25, *P* = 0.88), plant species and season significantly influenced the observed changes in bacterial presence (Figure 3, *F*_3,272_ = 4.56, *P* = 0.004 and *F*_2,272_ = 210.8, *P* < 0.001, respectively). The two pioneer plants, *T. farfara* and *S. caprea*, harbored higher 16S rDNA copy numbers for bacterial endophytes compared to *C. epigejos* and *P. abies* (*F*_2,272_ = 1.90, *P* = 0.001). Bacterial copy numbers in young leaves at the start of the growing season varied significantly among the plant species studied (Figure 3). *Salix caprea*, *T. farfara*, and *C. epigejos*, which lose their leaves in autumn and grow new leaves in spring, had initial bacterial copy numbers starting at the detection limit of the qPCR method in some individuals. The bacterial copy numbers in the overwintering needles of *P. abies* were higher, but also significantly lower in spring than in autumn, implying marked die‐out of the endophytic bacteria during the winter. The summer colonization levels of endophytic bacteria did not differ significantly from those in autumn, suggesting that colonization levels plateau during the summer months (up to 3.6577 × 10^7^ 16S rDNA copies ng DNA^–1^, *S. caprea*) and remain relatively stable until leaves begin to senesce. Overall, bacterial copy numbers were positively correlated with total C content in leaves during the spring (Table 2, *r* = 0.21, *P* = 0.03), contrary to the summer and autumn samplings, when this relationship was negative (Table 2, C:N_summer_: *r* = –0.47, *P* = 0.001; C:N_autumn_: *r* = –0.45, *P* < 0.001; C:P_autumn_: *r* = –0.24, *P* = 0.02). A significant negative relationship between bacterial copy numbers and leaf N content was observed in autumn *P. abies* samples (Appendix S5).

**Table 2:** Pearson correlation coefficient values for C, N, and P composition of leaf tissue (total C, N, P; C:N, C:P, and N:P), and bacterial (16S rDNA) and fungal (β‐actin) copy number, and fungi: bacteria ratio in the whole dataset from the Sokolov chronosequence, Czech Republic, during the spring, summer, and autumn sampling events.

Leaf	Bacterial copy number			Fungal copy number			Ratio of fungal to bacterial copy number		
chemistry	Spring	Summer	Autumn	Spring	Summer	Autumn	Spring	Summer	Autumn
C	**0.21**	–0.05	**–0.45**	**0.51**	**0.41**	**0.50**	0.11	**0.26**	**0.59**
N	–0.17	**0.51**	**0.34**	0.04	–0.07	0.01	0.15	**–0.50**	**–0.22**
P	–0.12	0.18	0.09	**0.22**	–0.17	–0.05	**0.22**	**–0.25**	–0.09
C:N	0.19	**–0.47**	**–0.45**	0.01	0.15	0.15	–0.14	**0.50**	**0.39**
C:P	0.14	–0.17	**–0.24**	–0.17	**0.24**	**0.21**	**–0.21**	**0.28**	**0.28**
N:P	–0.05	**0.39**	0.21	**–0.30**	0.09	0.06	–0.15	**−0.30**	−0.10

*Note*: Bold values are significant correlations (*P* < 0.05).

#### Fungal endophytes

**Figure 4:**
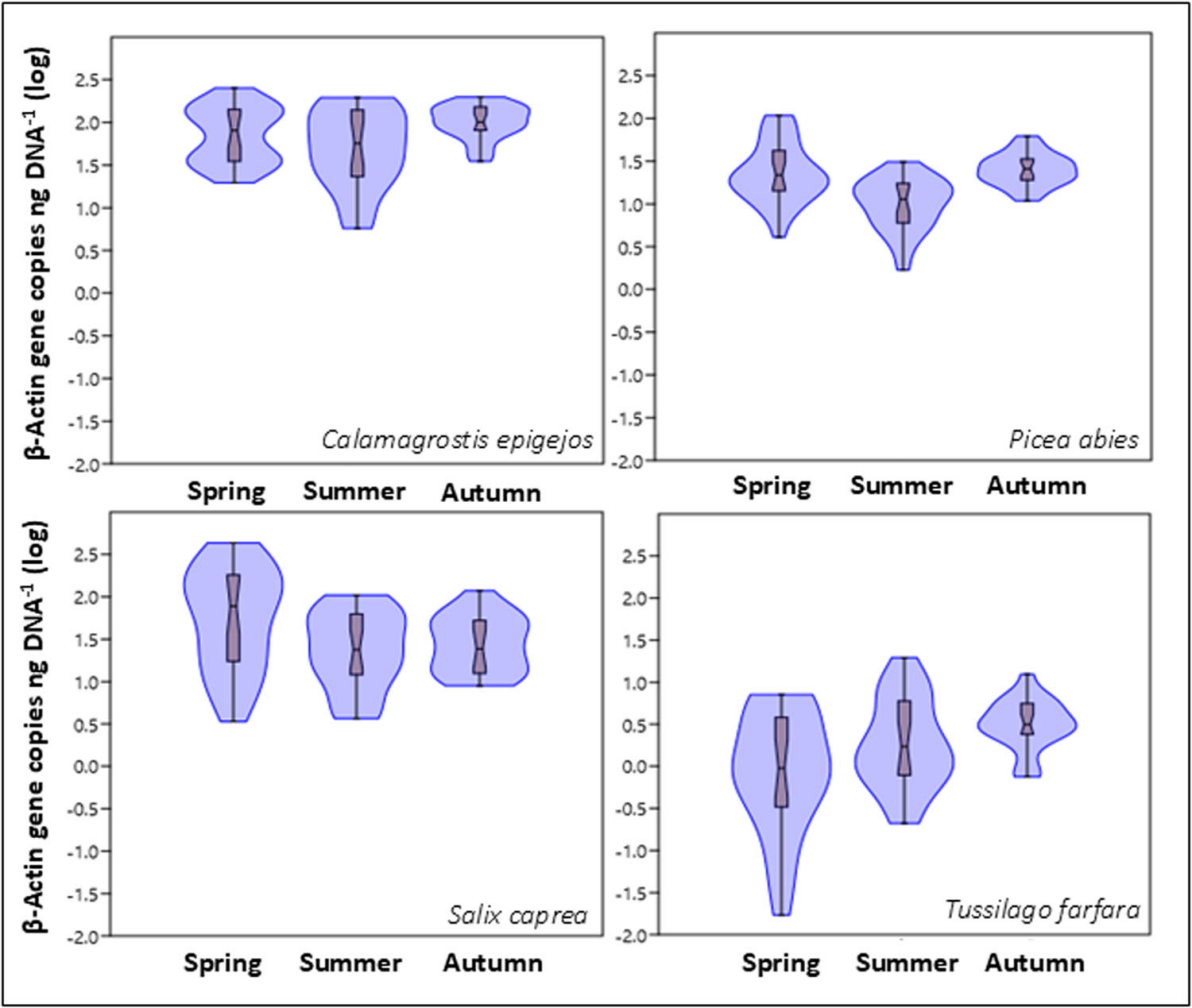
Violin plots showing the level of fungal endophytes (as β‐actin gene copies ng DNA^–1^) in leaves from four plant host species (*Calamagrostis epigejos*, *Picea abies*, *Salix caprea*, and *Tussilago farfara*) from the experimental locations along a chronosequence gradient, Sokolov, Czech Republic, at three sampling times in the growing season (spring, summer, autumn). Values were log‐transformed (base 10); box plots display the median (central constriction), interquartile range (upper and lower box borders), lower/upper adjacent values (black lines), and outliers (open circles and stars).

Fungal leaf endophytes, estimated as the number of β‐actin gene copies normalized per nanogram of total extracted DNA, ranged between 0.02 and 432 β‐actin copies per ng DNA, with high variability throughout the data set (Appendix S2). Unlike in bacteria, the overall springtime fungal copy numbers were comparable to those later in the year (Figure 4), while in some species (*S. caprea* and *P. abies* at locations I and II and *C. epigejos* at location II), fungal colonization was highest in the spring. Plant species significantly influenced the observed changes in fungal copy numbers (*F*_3,272_ = 2.55, *P* = 0.05). Compared to the other three species, *T. farfara* had significantly fewer copies of β‐actin per ng DNA (Figure 4, *P* < 0.001). Neither the location/successional stage nor the season had a significant effect (*F*_3,272_ = 2.55, *P* = 0.05 and *F*_3,272_ = 2.36, *P* = 0.09, respectively). Overall, fungal copy numbers were positively correlated with total C content in the leaf tissue throughout the year (Table 2, C_spring_: *r* = 0.51, *P* < 0.001; C_summer_: *r* = 0.41, *P* < 0.001; C_autumn_: *r* = 0.50, *P* < 0.001).

**Figure 5:**
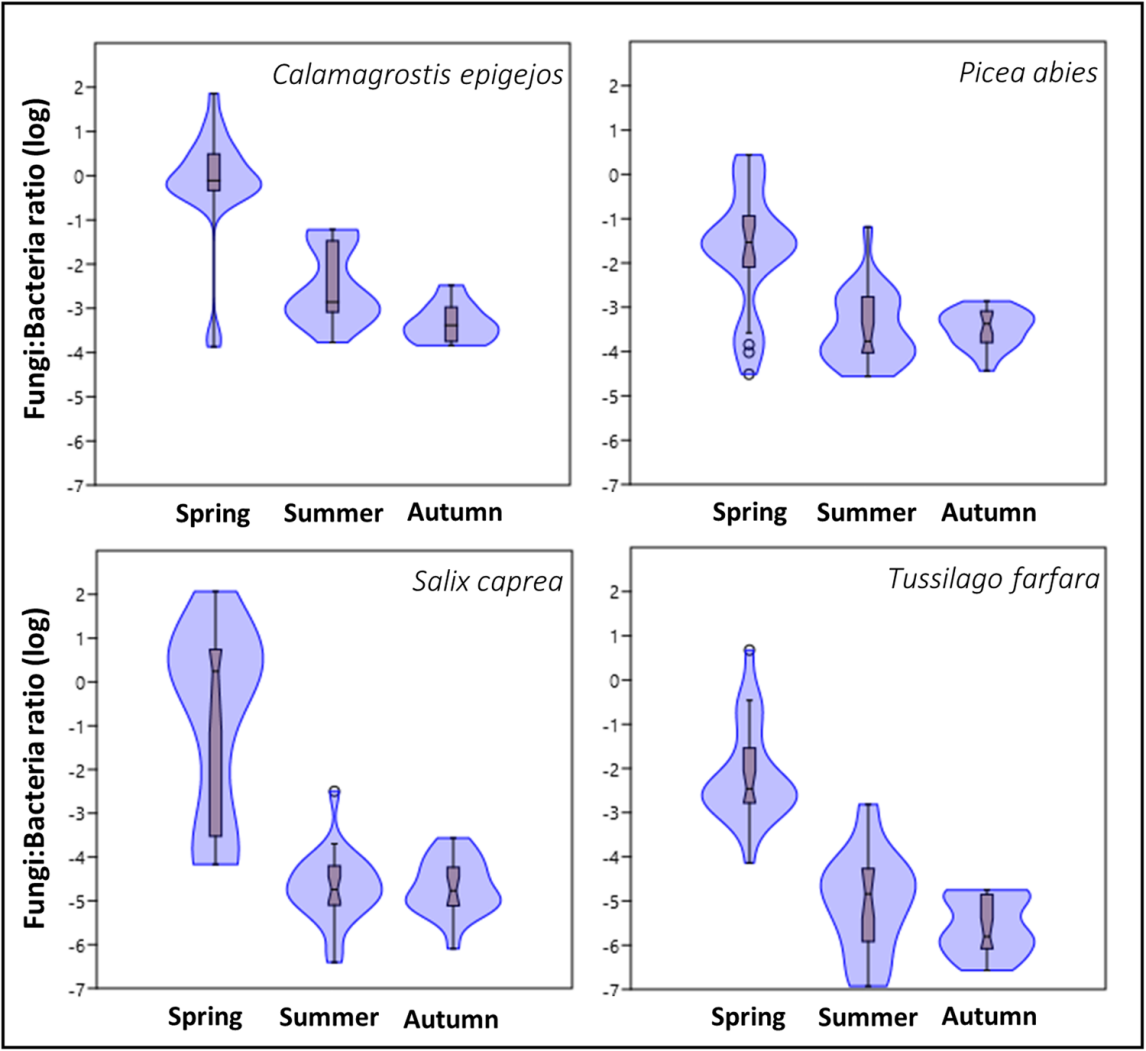
Violin plots showing ratio of fungi to bacteria in the leaves of four plant host species (*Calamagrostis epigejos*, *Picea abies*, *Salix caprea*, and *Tussilago farfara*) from the experimental locations along a chronosequence gradient, Sokolov, Czech Republic, at three sampling times in a growing season (spring, summer, and autumn). Values were log‐transformed (base 10), box plots display the median (central constriction), interquartile range (upper and lower box borders), lower/upper adjacent values (black lines), and outliers (open circles and stars).

Ratios of fungal to bacterial copy numbers (F:B) were significantly affected by plant species and season (Figure 5, *F*_3,272_ = 19.28, *P* < 0.001 and *F*_2,272_ = 134.2, *P* < 0.001, respectively), while we found no significant effect of the location or successional stage (*F*_3,292_ = 1.1, *P* < 0.34). In general, the springtime F:B ratios were strikingly higher compared to those later in the season.

**Figure 6:**
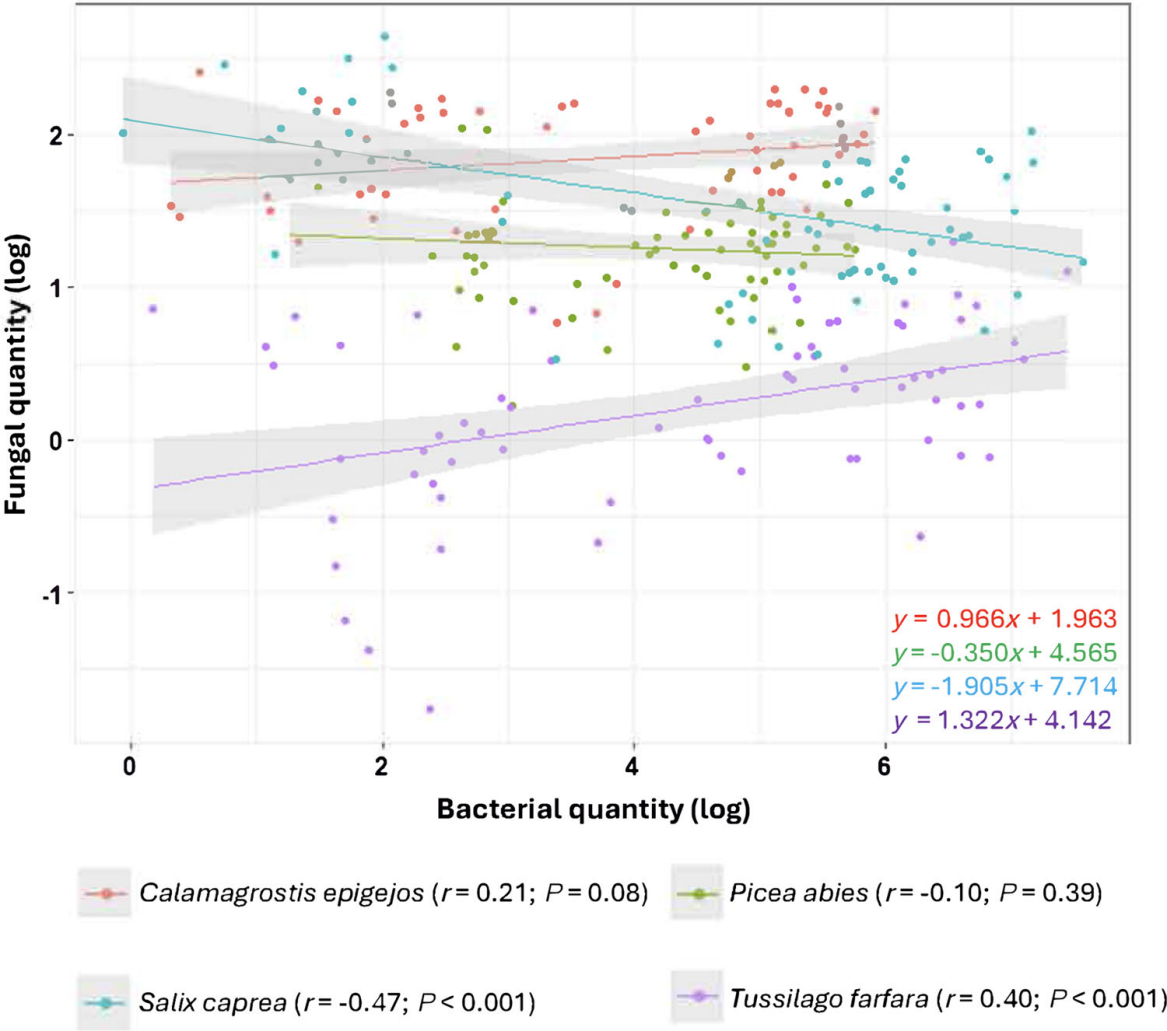
Scatter plots of Pearson correlation between the qPCR‐based estimates of fungal and bacterial levels (β‐actin gene copies ng DNA^–1^ and 16S rDNA gene copies ng DNA^–1^, respectively) in the foliar endosphere of four plant hosts (*Calamagrostis epigejos*, *Picea abies*, *Salix caprea*, and *Tussilago farfara*), from Sokolov experimental locations, Czech Republic. Values were log‐transformed (base 10); plots, Pearson's *r*, *P* values, and regression equations correspond in color to the plant host.

The fungal and bacterial copy numbers were correlated in each of the two pioneer plants (Figure 6), but the correlation was negative in *S. caprea* (*r* = –0.47, *P* < 0.001) and positive in *T. farfara* (*r* = 0.40, *P* < 0.001). When we analyzed the entire data set, fungal and bacterial copy numbers were not correlated in spring (*P* = 0.29) or summer leaves (*P* = 0.46), but they were negatively correlated in autumn samples (Appendix S6, *r* = –0.29, *P* < 0.001).

## DISCUSSION

### Experimental validation of the qPCR‐based assay for quantifying endophytic levels

In this study, we confirmed the usefulness of the bacteria‐specific 16S primer pair 335F/769R and the primer set targeting fungal β‐actin in quantifying bacterial and fungal endophytes by qPCR in leaf samples with high host plant DNA content. We believe our results are more precise than the other available options discussed in the introduction and provide a useful measure to describe the overall levels of bacterial and fungal endophytes in the plant endosphere. However, we deliberately avoided using the term “abundance” throughout the text, as the gene copy numbers cannot be directly linked to cell number, and, hence, biomass. Rather, we opted for the terms amount or quantity, level, copy number, and colonization.

Both tested primer sets (335F/769R and ACT‐512F/ACT‐783R) performed well in avoiding the major pitfalls of the method—the amplification of non‐target DNA by the universal 16S primer, and the overestimation caused by multiple gene copies of commonly used fungal target genes. However, fewer limitations to the approach remain. Although the variation is substantially smaller compared to the fungal rDNA (ranging from an estimated 14 to 1442 copies, mean = 113, median = 82; Lofgren et al., 2018), the 16S rRNA gene may also be present in multiple copies in bacterial genomes (bacteria tend to have fewer than 7 rDNA copies, median = 5; Lavrinienko et al., 2021). It would therefore be optimal to use a single‐copy bacterial gene target for amplification in the qPCR assay. Although previous studies have identified many single‐copy genes in bacterial genomes, there is still no consensus or enough experimental data available on their universality or usefulness as markers (Wang et al., 2022). However, because the variation in rDNA copy number seems to be a greater problem for studies of microbial eukaryotes than prokaryotes (Lavrinienko et al., 2021), we believe the bacteria‐specific primers targeting the 16S rDNA currently represent the most accurate option available to quantify the levels of colonization by bacterial endophytes. Both primers tested here have been shown to have broad coverage (Caporaso et al., 2011; Nakano, 2018), and both 16S rDNA and β‐actin genes are commonly used as phylogenetic markers (Stielow et al., 2015; Wang et al., 2022). However, knowledge of the assemblage composition in the studied system can help substantially with the sound interpretation of the quantification data, and we recommend this approach as a complement to NGS‐generated compositional data sets.

Further potential limitations that should be considered when using this approach to analyze complex environmental samples include those inherent to sample processing, DNA extraction, and the PCR approach itself. Surface‐sterilization methods should be optimized for specific plant species and the efficacy of removing surface contaminants should be balanced with minimizing the impact of sterilization on the DNA of endophytic microorganisms (Yu et al., 2022). There are significant differences among the available extraction methods in their efficiency in breaking up the cells of different organisms, extracting DNA from biological samples, and removing PCR inhibitors. Approaches optimized for plant tissues (Heikrujam et al., 2020) are recommended when working with foliar endophytes. As seen in our environmental data, microbial loads can vary substantially among different host species, but also among individuals of the same species. Furthermore, the plant endosphere can be expected to contain DNA from live, dead, and damaged microbial cells, as well as free DNA, and should be considered when interpreting qPCR data for endophytes. Although PCR is very specific, amplification artifacts can occur, and without amplicon verification, false positives can be interpreted as valid amplifications (Ruijter et al., 2019), which could specifically impact the analysis of plant tissues with very low levels of endophytes. However, simple tools such as agarose gel electrophoresis or the melting curve analysis can be used to confirm whether the fluorescence signal is generated from target templates or nonspecific PCR products (Smith and Osborn, 2009).

The qPCR approach relies on the use of an external standard to create calibration curves. The accuracy of the quantification therefore depends on the accuracy of the standard. Standard design, production, determination of the exact standard concentration, and its stability over long storage times should be given careful consideration as these can be problematic. Although significantly more costly, droplet digital PCR (ddPCR) is an alternative to conventional qPCR and offers several advantages, namely absolute quantification without the need for an external calibration curve, less susceptibility to PCR inhibitors and variation in PCR efficiency, and increased precision in low concentration samples (Taylor et al., 2017).

Another important issue to mention is normalization. How to normalize qPCR data in microbial ecology studies is a subject of long‐standing debate. In our case, the fresh mass to dry mass ratios of homogenized leaf material from the same host plant species were very similar. However, although the mass of tissue going into the DNA extraction was approximately equal, the amount of extracted DNA varied significantly between these samples (Appendix S2), which is typical for this kind of source material. We therefore normalized the qPCR results to the amount of total extracted DNA (mostly of plant origin), which we think is the correct way to allow meaningful between‐sample comparison in this type of study.

### Field assessment of colonization levels of foliar endophytes

One of the main aims of this study was to show that including colonization levels in ecological studies of foliar endophytes can add useful information and expand insights into plant–endophyte interactions.

The overall levels of bacterial endophytes in the four plant hosts studied here were found to be independent of the ecosystem stage and at the same order of magnitude in all four studied plant hosts. However, in the two pioneers, *S. caprea* and *T. farfara*, they were significantly higher than in *P. abies* and *C. epigejos*, possibly due to higher carbohydrate availability within the apoplast, provided by increased rates of photosynthesis per unit of leaf area typically observed in pioneer species (Bazzaz and Pickett, 1980; Barau et al., 2015, and references therein). We detected a positive correlation between the 16S rDNA gene copy numbers and total C content in leaf tissue during the spring sampling when leaves generally contain the lowest ratios of complex structural C compounds to simple, bioavailable, nonstructural carbohydrates (Martínez‐Vilalta et al., 2016; Liu et al., 2019). Because organic C limitation generally represents the most important environmental constraint on bacterial growth (Soong et al., 2020 and references therein), it may be one of the key factors that facilitate greater colonization extent in foliar endophytic bacteria in pioneer species and warrants further detailed study to confirm this hypothesis. Similar to colonization by bacteria, fungal endophytic colonization extent was unaffected by ecosystem age, but more host‐specific compared to bacterial endophytes, and with greater interspecific variation. The highest fungal copy numbers were observed in the grass, *C. epigejos*, followed by *S. caprea*, *P. abies*, and finally, *T. farfara*.

The relationships between the levels of colonization by both bacterial and fungal endophytes and foliar tissue stoichiometry were highly specific for host species and endophyte group, and the results did not show any generalizable patterns (for summary, see Appendix S2). For example, the copy numbers for bacterial endophytes in spring leaves of *S. caprea* were negatively correlated with both N and P content in leaves, while for fungi, this relationship was positive. Later in the year, however, these relationships in both endophyte groups became either weaker, or nonsignificant. The virtually complete decoupling of the relationship between foliar stoichiometry and endophyte levels at the end of the growing season was observed in all studied plant host species and likely reflects species‐specific dynamic changes in the apoplast during leaf senescence (Borniego et al., 2020 and references therein). The overall levels of bacterial and fungal endophytes (or their ratios) in autumn samples did not differ from the summer ones. This result may indicate that, in general, virtually all available space in the foliar endosphere is colonized by endophytic microbes by summer and that these colonization levels are maintained until the end of the growing season.

While roots and the rhizosphere are interfaces where plant hosts actively modify the associated microbial communities both quantitatively and qualitatively via changes in root size and architecture, altered exudation patterns, or direct mycorrhizal effects (Bednarek et al., 2010), the aboveground organs are thought of as inflexible in terms of such extensive physiological changes. However, our quantitative data indicate that the host‐specific endosphere environment does shape colonization levels by foliar bacterial and fungal endophytes in a significant way, although the effect on the two groups is different. Based on the results of this study, we argue that quantifying colonization levels can provide important new insights into endophytic ecology and function and that the qPCR‐based quantification of colonization levels represents a high‐throughput, low‐cost approach that can, despite its limitations described above, significantly improve the interpretation of descriptive data generated, for example, by next‐generation sequencing.

## AUTHOR CONTRIBUTIONS

D.S. and C.C.P.P. conceived the hypotheses and designed methodology; C.C.P.P. and D.S. collected the samples and data; J.Ba. tested and optimized the methodology for DNA extraction and PCR; J.F. selected experimental locations and plant host species; J.Bor. optimized and conducted chemical analyses; P.R. optimized the epifluorescence microscopy protocol and provided microscopic count data; C.C.P.P., D.S. analyzed data, interpreted results, and drafted the manuscript; J.Ba., J.Bor., and J.F. contributed critically to data analysis and interpretation and to drafting the final text.

## CONFLICT OF INTEREST STATEMENT

The authors declare no conflict of interest.

## Supporting information


**Appendix S1.** qPCR melting curves (A) and agarose gel electrophoresis results for 18S and β‐actin amplicons (B) from the analysis of samples of standard mock communities.

**Appendix S2.** Sample information (location, season, and plant species), chemical composition of leaf tissue (carbon, nitrogen, and phosphorus), molar ratios (C:N, C:P, and N:P), concentration of extracted DNA per sample, and bacterial and fungal rDNA copy numbers.

**Appendix S3.** Bacterial quantification (16S rDNA copy number) using different primer sets (512F/783R; 335F/769R) and nine samples containing a mock community (MC), algal DNA from *Chlorella vulgaris* (Chl), and a mix of both, at different concentrations.

**Appendix S4.** Fungal quantification of the mock communities using microscopy and qPCR of three different target fungal genes (18S, ITS, and β‐actin).

**Appendix S5.** Pearson correlation coefficient values for the chemical composition of leaf tissue (total carbon, nitrogen, phosphorus; carbon to nitrogen ratio [C:N], carbon to phosphorus ratio [C:P], and nitrogen to phosphorus ratio [N:P]) and bacterial (16S rDNA) and fungal (β‐actin) copy number, and fungi to bacteria ratio in the four experimental plant hosts (*Calamagrostis epigejos*, *Picea abies*, *Salix caprea*, and *Tussilago farfara*).

**Appendix S6**. Scatter plots showing the Pearson correlation between the qPCR‐based estimates of the total extent of fungal and bacterial colonization (β‐actin gene copies ng DNA^–1^ and 16S rDNA gene copies ng DNA^–1^, respectively) colonizing the foliar endosphere of the four studied plant hosts from the experimental locations along a chronosequence gradient, Sokolov, Czech Republic, at three sampling points during a single growing season (spring, summer, and autumn).

## Data Availability

Relevant background data are available as supporting material.
