# Supplementary figures and images for: qPCR‐based quantification reveals high plant host‐specificity of endophytic colonization levels in leaves
Source: Am J Bot. 2024 Dec 16;112(1):e16448. doi: 10.1002/ajb2.16448 (PMC11744438; doi:10.1002/ajb2.16448)

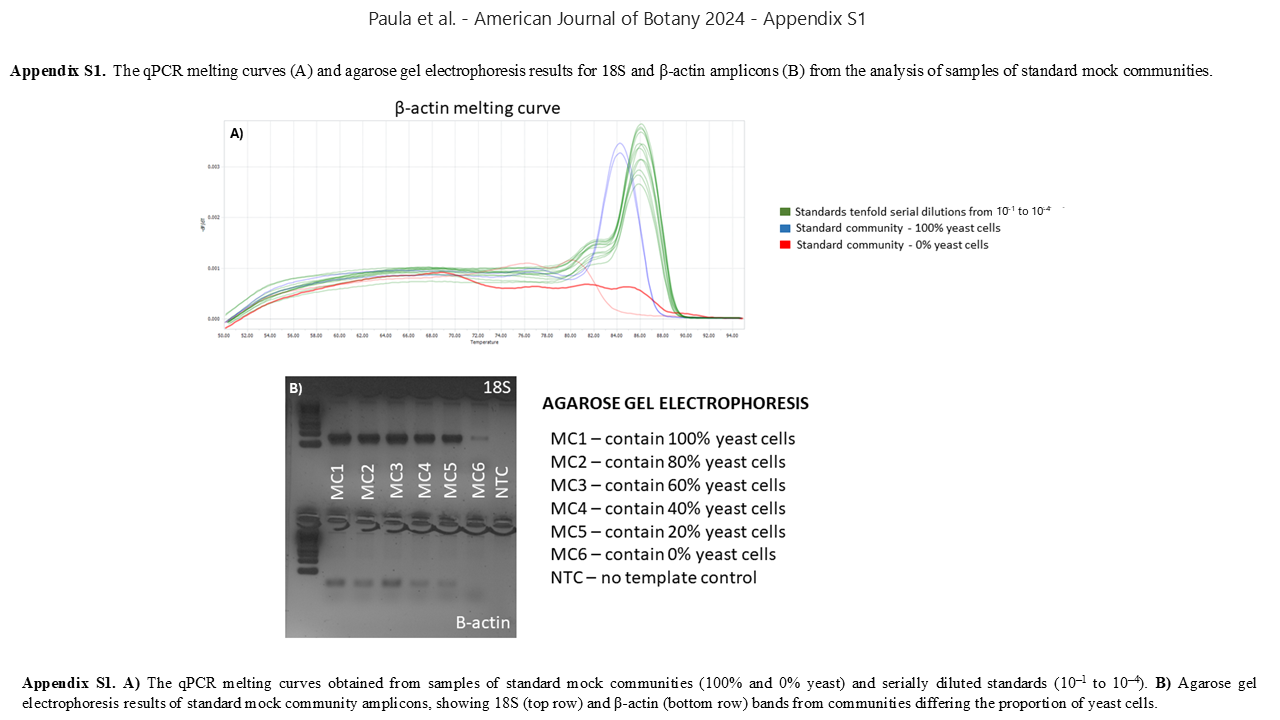

Supplement: Supplementary file 1 — Appendix S1. qPCR melting curves (A) and agarose gel electrophoresis results for 18S and β‐actin amplicons (B) from the analysis of samples of standard mock communities. [file AJB2-112-e16448-s001.tif]
