# Supplementary material for: qPCR‐based quantification reveals high plant host‐specificity of endophytic colonization levels in leaves
Source: Am J Bot. 2024 Dec 16;112(1):e16448. doi: 10.1002/ajb2.16448 (PMC11744438; doi:10.1002/ajb2.16448)
Supplement: Supplementary file 3 — Appendix S3. Bacterial quantification (16S rDNA copy number) using different primer sets (512F/783R; 335F/769R) and nine samples containing a mock community (MC), algal DNA from Chlorella vulgaris (Chl), and a mix of both, at different concentrations. [file AJB2-112-e16448-s003.docx]

Paula et al.—American Journal of Botany 2024—Appendix S3

**Appendix S3.** Bacterial quantification (16S rDNA copy number) using different primer sets (512F/783R; 335F/769R) and nine samples containing a mock community (MC), algal DNA from Chlorella vulgaris (Chl), and a mix of both, at different concentrations. Averages ± standard deviation are shown.

|  | **PRIMERS SET** | |
| --- | --- | --- |
| **SAMPLES** | **512F/783R** | **335F/769R** |
| **MC (0,01ng gDNA)** | 10,485,740,000 ± 1,633,875,000 | 4,584,381,000 ± 476,456,400 |
| **MC (0,1ng gDNA)** | 5,411,406,000 ± 708,874,000 | 3,387,421,000 ± 41,685,850 |
| **MC : Chl (100 : 1)** | 1,422,274,000 ± 308,926,700 | 1,444,878,000 ± 42,307,230 |
| **MC : Chl (10 : 1)** | 1,796,244,000 ± 267,007,400 | 1,275,966,000 ± 36,092,410 |
| **MC : Chl (1 : 1)** | 992,636,000 ± 296,627,800 | 712,957,100 ± 53,395,660 |
| **MC : Chl (1 : 10)** | 370,707,400 ± 6,587,615 | 153,769,900 ± 146,852,400 |
| **MC : Chl (1 : 100)** | 125,714,100 ± 1,436,603 | 22,048,920 ± 1,756,188 |
| **Chl (0,1ng gDNA)** | 553,198,200 ± 13,956,910 | - |
| **Chll (0,01ng gDNA)** | 3,587,330,000 ± 11,387,690 | - |
