# Supplementary material for: qPCR‐based quantification reveals high plant host‐specificity of endophytic colonization levels in leaves
Source: Am J Bot. 2024 Dec 16;112(1):e16448. doi: 10.1002/ajb2.16448 (PMC11744438; doi:10.1002/ajb2.16448)
Supplement: Supplementary file 5 — Appendix S5. Pearson correlation coefficient values for the chemical composition of leaf tissue (total carbon, nitrogen, phosphorus; carbon to nitrogen ratio [C:N], carbon to phosphorus ratio [C:P], and nitrogen to phosphorus ratio [N:P]) and bacterial (16S rDNA) and fungal (β‐actin) copy number, and fungi to bacteria ratio in the four experimental plant hosts (Calamagrostis epigejos, Picea abies, Salix caprea, and Tussilago farfara). [file AJB2-112-e16448-s002.docx]

Paula et al.—American Journal of Botany 2024—Appendix S5

**Appendix S5.** Pearson correlation coefficient values for the chemical composition of leaf tissue (total carbon, nitrogen, phosphorus; carbon to nitrogen ratio [C:N], carbon to phosphorus ratio [C:P], and nitrogen to phosphorus ratio [N:P]) and bacterial (16S rDNA) and fungal (β-actin) copy number, and fungi to bacteria ratio in the four experimental plant hosts (Calamagrostis epigejos, Picea abies, Salix caprea, and Tussilago farfara). Leaves were sampled three times during a single growing season (spring, summer, and autumn).

|  | **Plant species** | **Season** | **C** | **N** | **P** | **C:N** | **C:P** | **N:P** |
| --- | --- | --- | --- | --- | --- | --- | --- | --- |
| **16S rDNA copy**  **ng DNA^-1^** | *Calamagrostis epigejos* | Spring | - | 0.524 | 0.585 | - 0.534 | - 0.517 | - |
|  |  | Summer | - | - | - | - | - | - |
|  |  | Autumn | - | - | - | - | - | - |
|  | *Picea abies* | Spring | - | - | - | - | - | - |
|  |  | Summer | 0.477 | - | - | - | - | - |
|  |  | Autumn | - | - 0.468 | - | - | - | - |
|  | *Salix caprea* | Spring | - | - 0.550 | - 0.461 | - | - | - |
|  |  | Summer | - 0.429 | - | - | - | - | - |
|  |  | Autumn | - | - | - | - | - | - |
|  | *Tussilago farfara* | Spring | - 0.546 | - | - | - | - | - |
|  |  | Summer | 0.436 | - | - | - | 0.436 | - |
|  |  | Autumn | - | - | - | - | - | - |
| **β-actin copy**  **ng DNA*^-1^*** | *Calamagrostis epigejos* | Spring | - | - | - | - | - | - |
|  |  | Summer | - | 0.511 | - | - 0.571 | - | - |
|  |  | Autumn | 0.459 | - | - | - | - | - |
|  | *Picea abies* | Spring | 0.459 | - | - | - | - | - |
|  |  | Summer | 0.417 | - | - | - | - 0.565 | - |
|  |  | Autumn | - | - | - | - | - | - |
|  | *Salix caprea* | Spring | - 0.644 | 0.728 | 0.701 | - 0.770 | - 0.802 | - |
|  |  | Summer | - | 0.444 | - | - 0.439 | - | - |
|  |  | Autumn | - | - | - | - | - | - |
|  | *Tussilago farfara* | Spring | - | - 0.523 | - | 0.476 | - | - 0.432 |
|  |  | Summer | - | - | - | - | - | - |
|  |  | Autumn | - | - | - | - | - | - |
| **Fungi:Bacteria**  **ratio** | *Calamagrostis epigejos* | Spring | - | - 0.461 | - 0.575 | 0.473 | 0.516 | - |
|  |  | Summer | - | 0.462 | - | - | - | - |
|  |  | Autumn | - | - | - | - 0.476 | - | - |
|  | *Picea abies* | Spring | 0.462 | - | - | - | - | - |
|  |  | Summer | - | - | - | - | - | - |
|  |  | Autumn | - | 0.659 | - | - 0.579 | - 0.548 | - |
|  | *Salix caprea* | Spring | 0.435 | 0.638 | 0.559 | - 0.705 | - 0.663 | - |
|  |  | Summer | - | - | - | - | - | - |
|  |  | Autumn | - | - | - | - | - | - |
|  | *Tussilago farfara* | Spring | - | - | - | - | - | - 0.410 |
|  |  | Summer | - 0.436 | - | - | - | - | - 0.483 |
|  |  | Autumn | - | - | - | - | - | - |
