# Supplementary material for: qPCR‐based quantification reveals high plant host‐specificity of endophytic colonization levels in leaves
Source: Am J Bot. 2024 Dec 16;112(1):e16448. doi: 10.1002/ajb2.16448 (PMC11744438; doi:10.1002/ajb2.16448)
Supplement: Supplementary file 6 — Appendix S6. Scatter plots showing the Pearson correlation between the qPCR‐based estimates of the total extent of fungal and bacterial colonization (β‐actin gene copies ng DNA–1 and 16S rDNA gene copies ng DNA–1, respectively) colonizing the foliar endosphere of the four studied plant hosts from the experimental locations along a chronosequence gradient, Sokolov, Czech Republic, at three sampling points during a single growing season (spring, summer, and autumn). [file AJB2-112-e16448-s005.pdf]

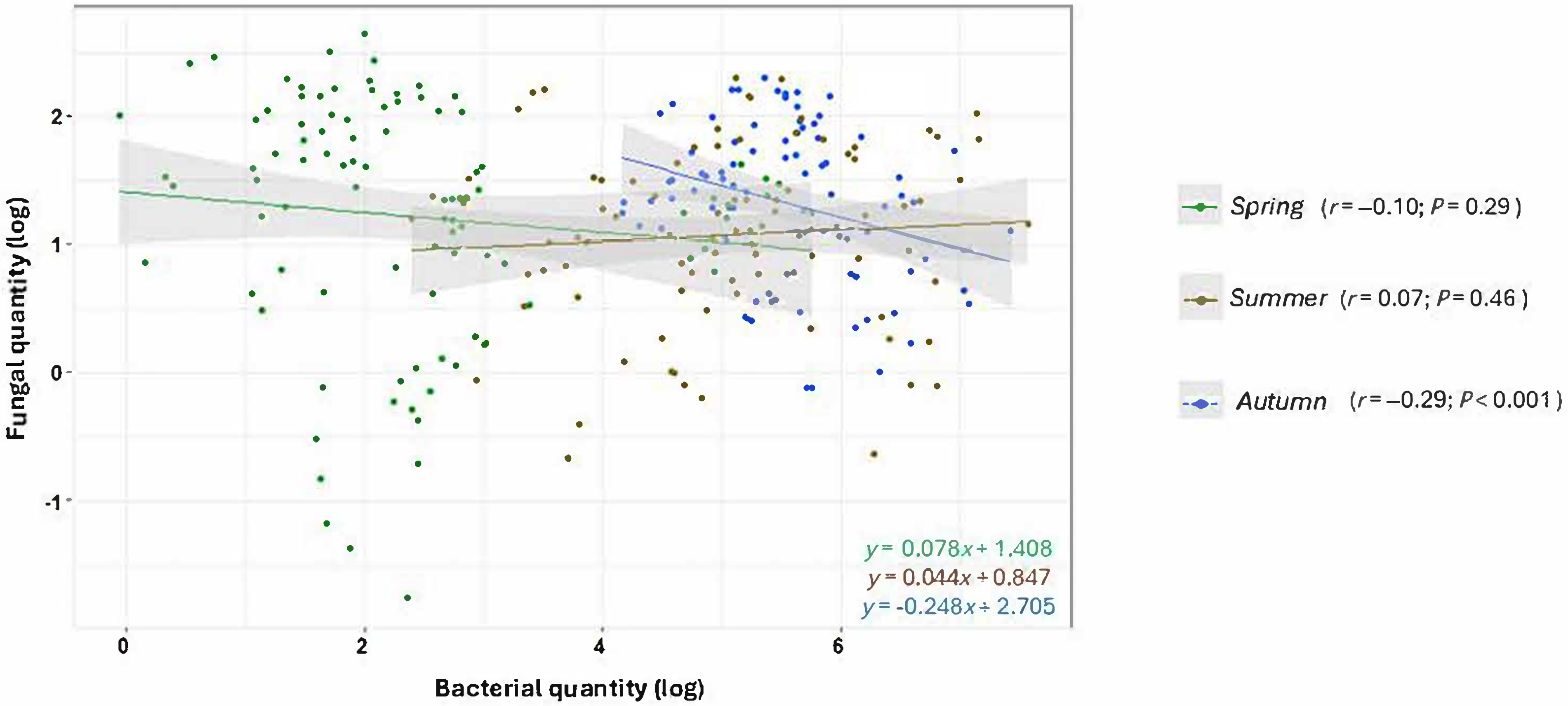

Appendix S6. Scatter plots showing the Pearson correlation between the qPCR-based estimates of the total extent of fungal and bacterial colonization (β-actin gene copies ng DNA^{-1} and 16S rDNA gene copies ng DNA^{-1}, respectively) colonizing the foliar endosphere of the four studied plant hosts (*Calamagrostis epigejos*, *Picea abies*, *Salix caprea*, and *Tussilago farfara*) from the experimental locations along a chronosequence gradient, Sokolov, Czech Republic, at three sampling points during a single growing season (spring, summer, and autumn). Values were log-transformed (base-10), Pearson's r values, P values, and the regression function correspond in color to that of the respective plant hosts.
